# Age and sex differences in the association between APOE genotype and Alzheimer’s disease in a Taiwan Chinese population

**DOI:** 10.3389/fnagi.2023.1246592

**Published:** 2023-08-23

**Authors:** Ling-Chun Huang, Mei-Yueh Lee, Ching-Fang Chien, Yang-Pei Chang, Kuan-Ying Li, Yuan-Han Yang

**Affiliations:** ^1^Department of Neurology, Kaohsiung Municipal Ta-Tung Hospital, Kaohsiung Medical University Hospital, Kaohsiung, Taiwan; ^2^Department of Neurology, Kaohsiung Medical University Hospital, Kaohsiung Medical University, Kaohsiung, Taiwan; ^3^Neuroscience Research Center, Kaohsiung Medical University, Kaohsiung, Taiwan; ^4^Division of Endocrinology and Metabolism, Department of Internal Medicine, Kaohsiung Medical University Hospital, Kaohsiung, Taiwan; ^5^Faculty of Medicine, College of Medicine, Kaohsiung Medical University, Kaohsiung, Taiwan; ^6^School of Post-Baccalaureate Medicine, College of Medicine, Kaohsiung Medical University, Kaohsiung, Taiwan

**Keywords:** Alzheimer’s disease, *apolipoprotein* E gene, genetic association, sex and age stratified analyses, Taiwan Chinese population

## Abstract

**Introduction:**

The *Apolipoprotein E* (*APOE*) epsilon (ε) 4 allele is a well-established risk factor for late-onset Alzheimer’s disease (AD). Reports on white ancestry populations have showed that age, sex, and ethnicity have different effects on the association between APOE genotype and AD. However, studies on Asian populations such as Taiwan Chinese populations are limited. This study aimed to evaluate the association between *APOE* genotype and AD in a Taiwan Chinese population, and to explore if the association varies by age and sex.

**Methods:**

We conducted a case-control study in 725 patients with AD and 1,067 age- and sex- matched controls without dementia from a Taiwan Chinese population. Logistic regression models were used to test the association between AD and *APOE* genotypes. Secondary analyses considered age (<75 or ≥75 years old), and sex stratified models.

**Results:**

The risk of AD was significantly increased for people with at least one copy of *APOE* ε4 (OR = 2.52, 95% CI = 2.01–3.17, *p* < 0.001) and in a dose-dependent manner. Our results did not show an statistically significance different in AD risk when women and men carrying APOEε4 were compared. Despite not reaching statistical significance, the risk of *APOE* ε4 for AD was higher among younger participants (OR = 3.21, 95% CI = 2.26–4.56, *p* < 0.001) compared to older ones (OR = 2.13, 95% CI = 1.53–2.97, *p* < 0.001). When considering both sex and age, the risk of AD was higher among older men carrying *APOE* ε4 (OR = 2.64, 95% CI = 1.51–4.60 in men; OR = 1.90, 95% CI = 1.26–2.86 in women), while women carrying *APOE* ε4 appeared to have an increased risk at a younger age (OR = 3.29, 95% CI = 2.20–4.93 in women; OR = 2.91, 95% CI = 1.40–6.05 in men).

**Discussion:**

The *APOE* ε4 allele represents a major risk factor for AD in the Taiwanese population. The effect of *APOE* ε4 allele on AD risk appeared to be stronger among men aged 75 years or more and among younger women.

## Introduction

Alzheimer’s disease (AD) is the leading cause of dementia in elderly individuals (Alzheimers Dement, 2020). Polymorphism in the *apolipoprotein E* (*APOE*) gene is a major risk determinant of late-onset AD (Farrer et al., 1997; Liu et al., 2013). Of the three major *APOE* allelic variants, epsilon (ε) 2, ε3 and ε4, *APOE* ε4 is associated with an increased risk, while *APOE* ε2 has been reported as having a protective effect over the risk of AD (Saunders et al., 1993; Corder et al., 1994; Farrer et al., 1997; Liu et al., 2013). Having a single *APOE* ε4 allele increases the risk of AD onset 2–4 fold and having two *APOE* ε4 alleles increases the risk about 8–12 fold (Farrer et al., 1997). Increasing evidence suggests that the effect of *APOE* ε4 on AD risk is exerted through inhibition of amyloid-β (Aβ) clearance and promotion of Aβ aggregation (Liu et al., 2013; Yamazaki et al., 2019). *APOE* ε4 also contributes to AD pathogenesis by impairing microglial responsiveness, lipid transport, synaptic integrity and plasticity, glucose metabolism, and cerebrovascular integrity and function (Liu et al., 2013; Yamazaki et al., 2019).

The risk conferred by *APOE* ε4 varies by age and sex, and these differences in AD risk have important implications for treatment trials, diagnostics, and therapeutics (Ungar et al., 2014). *APOE* ε4 exerts its maximal effect on AD risk by the early 70’s, with a reduction in risk after age 85 in both sexes (Jarvik et al., 1995; Farrer et al., 1997). Evidence indicates that the *APOE* ε4 risk for AD is greater in women than men (Payami et al., 1994; Farrer et al., 1997; Altmann et al., 2014; Buckley et al., 2018). Whereas Neu et al. (2017) found that men and women with the *APOE* ε4 genotype did not show a difference in AD risk across the age span of 55–85 years, but women had an increased risk between the ages of 65 and 75 (Neu et al., 2017).

The effect of ethnic background on the *APOE* association with AD risk has long been known, with African American and Hispanic *APOE* ε4 carriers having a lower risk than Caucasian *APOE* ε4 carriers, and Japanese carriers having the highest odd ratios (ORs) (Farrer et al., 1997; Tang et al., 1998). China has the largest population of patients with dementia in the world (Jia et al., 2020). The associations between *APOE* genotype and AD risk in the Chinese population were reported (Liu et al., 2014; Wu et al., 2015; Chen et al., 2022), however, the effect of age and sex on *APOE* ε4 risk in Chinese population is still unknown. In addition, data on this in the Taiwan Chinese population remains limited due to small sample sizes in previous studies (Hong et al., 1996; Liu et al., 1999; Hu et al., 2000; Huang et al., 2002; Lai et al., 2003). Therefore, we conducted the current study to evaluate the association between *APOE* genotype and AD in a Taiwan Chinese population, and to explore if the association varies by age and sex.

## Materials and methods

### Subjects

The current study was part of a project by the Taiwan Precision Medicine Initiative (TPMI). The TPMI is a partnership between Academia Sinica and top medical centers in Taiwan to bring genetic information into clinical practice. It is utilizing big data analysis of the genetic and clinical information of a large cohort to accurately predict personal risk for common diseases, and TPMI aims to promote early disease screening, tailored medical treatment, and prevention in Taiwan. This is a case-control study and we recruited subjects at the outpatient departments of Kaohsiung Municipal Ta-Tung Hospital, Kaohsiung Municipal Hsiao-Kang Hospital and Kaohsiung Medical University Hospital. The enrollment period was from November 2019 to December 2021. A total of 725 clinically diagnosed AD patients were recruited. The diagnosis of AD was based on the National Institute of Neurological and Communicative Disorders and Stroke-Alzheimer’s Disease and Related Disorders Association criteria (McKhann et al., 1984). One thousand five hundred thirty-seven non-demented participants who visited the outpatient departments for subjective memory complaints other medical purposes were also recruited. After being age- and sex-matched with the AD group, 1,067 participants were assigned as the control group. All subjects in the control group were screened by an instrument of ascertainment of dementia 8 (AD8) (Yang et al., 2011) and had a score of <2 to exclude individuals with early stage dementia, or they were determined to have a Clinical Dementia Rating^®^ (Morris, 1993) score of zero as conducted by an experienced physician. All subjects enrolled in this study were Taiwanese. Subjects were examined for mutations of the amyloid precursor protein gene, presenilin-1 gene and presenilin-2 gene to exclude familial AD (Cruts et al., 1998). The participants and their relatives were informed of the details of the study. The Kaohsiung Medical University Hospital Institutional Review Board [KMUHIRB-SV(II)-20190059, KMUHIRB-SV(II)-20200034, KMUHIRB-E(II)-20220263 and KMUHIRB-SV(I)-20230025] approved the study protocol and the participants provided written informed consent prior to their inclusion.

### *APOE* genotype

The blood DNA samples from all subjects participating in the TPMI project with Academia Sinica were genotyped using the Axiom Genome-Wide TWB 2.0 Array Plate (Thermo Fisher Scientific, Waltham, MA, USA) (Wei et al., 2021). The data for the two *APOE* Single-Nucleotide Polymorphisms (SNPs) (rs429358 and rs7412) were exported using Axiom Analysis Suite (Thermo Fisher Scientific) and PLINK software (Purcell et al., 2007). The data for the two SNPs from a total of 1,792 samples from the AD and control groups were validated by TaqMan^®^ SNP Genotyping Assays (Thermo Fisher Scientific).

### Statistical analysis

Data were presented as the mean ± standard deviation or proportions. The χ^2^ test was used to compare categorical data (sex, *APOE* genotype and allele) and the *t*-test was used to compare continuous data (age) between AD and control groups. Due to the lower frequency of APOE ε2/ε2 and ε4/ε4 genotypes, we merged ε2/ε2 with the ε2/ε3 to form the ε2 group, and ε3/ε4 with the ε4/ε4 to form the ε4 group to test the effect of *APOE* genotype using the reference of ε3/ε3.

The association between AD and *APOE* was tested using logistic regression models, and age and/or sex were adjusted as appropriate. Secondary analyses considered age and sex stratified analyses. Age was dichotomized using 75 years of age as the threshold to define two different groups: younger (<75 years old) and older (≥75 years old) participants. To compare the differences in ORs among different age groups and sex, we analyzed the interactions between *APOE* and age, and *APOE* and sex, respectively. All analyses were performed using SPSS 26.0 (SPSS Inc., Chicago, IL, USA). A two-tailed *P*-value of <0.05 was considered to indicate a statistically significant difference.

## Results

### Demographic features of the study participants

Table 1 presents the demographic characteristics and *APOE* genotype of the participants. A total of 1,792 participants were recruited: 725 AD cases and 1,067 cognitively healthy individuals. The proportion of women was slightly higher among the AD cases compared to cognitively healthy participants (68 versus 66%, respectively). The average age of all participants was 75 years. As expected, the frequency of APOE ε4 was significantly higher among AD cases compared to cognitively healthy individuals (17.9 versus 8.2%, respectively).

**TABLE 1:** Demographic characteristics of the study participants.

Characteristic	AD (N = 725)	Control (N = 1,067)	P-value
Age (years)	75.1 ± 7.6	74.6 ± 5.9	0.113
Sex, women	490 (67.6)	706 (66.2)	0.531
≥1 copy of *APOE* ε4	233 (32.1)	169 (15.8)	<0.001
*APOE* genotype			<0.001
ε2/ε2	1 (0.1)	6 (0.6)	
ε2/ε3	64 (8.8)	140 (13.1)	
ε3/ε3	427 (58.9)	752 (70.5)	
ε3/ε4	206 (28.4)	162 (15.2)	
ε4/ε4	27 (3.7)	7 (0.7)	
**Allele**			
ε2	66 (4.6)	152 (7.1)	
ε3	1,124 (77.5)	1,806 (84.6)	
ε4	260 (17.9)	176 (8.2)	

Data are shown as the mean ± SD for quantitative variables and n (%) for qualitative variables. Age of AD group: age at clinical onset; Age of control group: age at recruitment. *p* < 0.05, statistically significant. AD, Alzheimer’s disease; *APOE*, apolipoprotein E; ε, epsilon.

### The association between Alzheimer’s disease and *APOE* ε4 allele and genotypes

As shown in Table 2, the risk of AD was significantly increased for carriers of at least one copy of *APOE* ε4 allele (OR = 2.52, 95% CI = 2.01–3.17). Homozygous individuals for the ε4 allele had an increased risk of developing AD (OR = 7.46, 95% CI = 3.22–17.30, *p* < 0.001) when compared to heterozygous carriers (OR = 2.32, 95% CI = 1.84–2.93, *p* < 0.001). Our findings indicate that *APOE* ε2 alleles confer a protective effect against the risk of AD, however, the results did not reach statistical significance. Using ε3/ε3 as the reference, the OR for *APOE* ε2/ε2 carriers is 0.26 (CI = 0.31–2.19, *p* = 0.216), and for ε2/ε3 carriers, the OR is 0.81 (CI = 0.59–1.12, *p* = 0.200).

**TABLE 2:** The association between Alzheimer’s disease and APOE ε4 allele and genotypes in Taiwanese participants.

	Odds ratio (95% CI)	P-value
≥1 copy of *APOE* ε4 allele	2.52 (2.01–3.17)	<0.001
ε4 heterozygous	2.32 (1.84–2.93)	<0.001
ε4 homozygous	7.46 (3.22–17.30)	<0.001
***APOE* genotype**		
ε3/ε3	1.00 (Reference)	.
ε2/ε2	0.26 (0.31–2.19)	0.216
ε2/ε3	0.81 (0.59–1.12)	0.200
ε3/ε4	2.24 (1.76–2.84)	<0.001
ε4/ε4	7.21 (3.11–16.76)	<0.001

Odds ratios adjusted by age and sex. *P* < 0.05, statistically significant. *APOE*, apolipoprotein E; ε, epsilon.

### Sex stratified association analyses

*Apolipoprotein E* ε4 carrier women (OR = 2.38, CI = 1.81–3.14) and men (OR = 2.64, CI = 1.75–3.98) did not show a significant difference in AD risk (*APOE*-sex interaction *P*-value = 0.46); however, *APOE* ε4 homozygous women appear to have an increased risk compared with homozygous men, but the number of ε4 homozygous cases is too small to demonstrate statistical differences between sexes (Table 3).

**TABLE 3:** The sex stratified association between Alzheimer’s disease and APOE ε4 allele and genotypes.

	Women (N = 1,196)		Men (N = 596)	
	Odds ratio (95% CI)	*P*-value	Odds ratio (95% CI)	*P*-value
≥1 copy of *APOE* ε4	2.38 (1.81–3.14)	<0.001	2.64 (1.75–3.98)	<0.001
ε4 heterozygous	2.11 (1.59–2.81)	<0.001	2.55 (1.67–3.88)	<0.001
ε4 homozygous	9.19 (3.04–24.84)	<0.001	4.73 (0.93–24.06)	0.061
***APOE* genotype**				
ε3/ε3	1.00 (Reference)	.	1.00 (Reference)	.
ε2/ε2, ε2/ε3	0.92 (0.63–1.35)	0.667	0.64 (0.36–1.13)	0.125
ε3/ε4, ε4/ε4	2.35 (1.77–3.12)	<0.001	2.48 (1.63–3.76)	<0.001

*APOE*-sex interaction *P*-value = 0.460. Odds ratios adjusted by age. *P* < 0.05, statistically significant. *APOE*, apolipoprotein E; ε, epsilon.

### Age stratified association analyses

The study participants were categorized into two different age groups using 75 years of age as the cutoff. As shown in Table 4, the risk of AD was increased among those carrying at least one copy of the *APOE* ε4 allele in both groups (OR = 2.23, 95% CI = 1.61–3.09 in the older group, OR = 3.33, 95% CI = 2.36–4.69 in the younger group). Despite not reaching statistical significance (*APOE*-age interaction *P*-value = 0.265), the *APOE* ε4 effect seems more evident at a younger age.

**TABLE 4:** The age stratified association between Alzheimer’s disease and APOE ε4 allele and genotypes.

	≥75 years (N = 922)		<75 years (N = 870)	
	Odds ratio (95% CI)	*P*-value	Odds ratio (95% CI)	*P*-value
≥1 copy of *APOE* ε4	2.23 (1.61–3.09)	<0.001	3.33 (2.36–4.69)	<0.001
ε4 heterozygous	2.11 (1.52–2.95)	<0.001	2.94 (2.06–4.21)	<0.001
ε4 homozygous	7.10 (1.46–34.50)	0.015	9.35 (3.34–26.18)	<0.001
***APOE* genotype**				
ε3/ε3	1.00 (Reference)	.	1.00 (Reference)	.
ε2/ε2, ε2/ε3	0.74 (0.47–1.14)	0.172	0.80 (0.49–1.29)	0.360
ε3/ε4, ε4/ε4	2.13 (1.53–2.97)	<0.001	3.21 (2.26–4.56)	<0.001

*APOE*-age interaction *P*-value = 0.265. Odds ratios adjusted by age and sex. P < 0.05, statistically significant. *APOE*, apolipoprotein E; ε, épsilon.

### Sex and age stratified association analyses

Stratifying by sex and age (Table 5), our results showed that in older age groups, *APOE* ε4 men carriers had a higher AD risk than women (OR = 2.64, 95% CI = 1.51–4.60 in men; OR = 1.90, 95% CI = 1.26–2.86 in women). Conversely, women carrying *APOE*ε4 had an increased risk at a younger age compared to men (OR = 3.29, 95% CI = 2.20–4.93 in women; OR = 2.91, 95% CI = 1.40–6.05 in men).

**TABLE 5:** The age and sex stratified association between Alzheimer’s disease and APOE ε4 allele and genotypes.

	≥75 years				<75 years			
	Women (*N* = 557)		Men (*N* = 365)		Women (*N* = 639)		Men (*N* = 231)	
	OR (95% CI)	*P*-value	OR (95% CI)	*P*-value	OR (95% CI)	*P*-value	OR (95% CI)	*P*-value
≥1 copy of *APOE* ε4	1.97 (1.32–2.95)	0.001	2.76 (1.60–4.78)	<0.001	3.29 (2.22–4.88)	<0.001	3.25 (1.59–6.67)	0.001
***APOE* genotype**								
ε3/ε3	1.00 (Reference)	.	1.00 (Reference)	.	1.00 (Reference)	.	1.00 (Reference)	.
ε2/ε2, ε2/ε3	0.77 (0.44–1.34)	0.767	0.70 (0.33–1.48)	0.351	1.01 (0.58–1.75)	0.940	0.49 (0.17–1.40)	0.184
ε3/ε4, ε4/ε4	1.90 (1.26–2.86)	0.002	2.64 (1.51–4.60)	0.001	3.29 (2.20–4.93)	<0.001	2.91 (1.40–6.05)	0.004

*APOE*-age-sex interaction *P*-value = 0.679. *P* < 0.05, statistically significant. *APOE*, apolipoprotein E; ε, epsilon.

## Discussion

We have demonstrated that the APOE ε4 allele represents a major risk factor for AD in the Taiwanese Chinese population, although its effect is weaker when compared to Caucasian populations. Although the difference did not reach a level of statistical significance, the risk appears to be higher in younger age groups. *APOE* ε4 carrier women and men did not show a significant difference in AD risk but *APOE* ε4 carrier men appear to have a higher risk at older age, while *APOE* ε4 carrier women had an increased risk at a younger age. While the impact of age and sex on the association between *APOE* genotype and AD is well-established in Caucasians, to the best of our knowledge, this is the first study in the Taiwan Chinese population.

*Apolipoprotein E* ε4 allele frequency in AD patients and the related risk for AD are different in people of different ethnicities. A previous study showed that *APOE* ε4 allele frequency in AD patients was highest in Caucasian (36.7%) followed by African American (32.2%), Japanese (27.8%) and Hispanic (19.2%) individuals (Farrer et al., 1997). Although *APOE* ε4 is considered the most important risk factor for AD, the *APOE* ε4 allele frequency in AD patients is lower in Taiwanese (17.9%) which was in line with previous studies (Liu et al., 1999; Huang et al., 2002). According to the study conducted by Farrer et al. (1997), the association between *APOE* ε4 and AD in Japanese people (ε3/ε4: OR 5.6, 95% CI = 3.9–8.0, ε4/ε4: OR 33.1, 95% CI = 13.6–80.5 relative to ε3/ε3) was higher than in Caucasian people (ε3/ε4: OR 2.7, 95% CI = 2.2–3.2, ε4/ε4: OR 12.5, 95% CI = 8.8–17.7), while the association was lower among African Americans (ε3/ε4: OR 1.1, 95% CI = 0.7–1.8, ε4/ε4: OR 5.7, 95% CI = 2.3–14.1) and Hispanics (ε3/ε4: OR 2.2, 95% CI = 1.3–3.4, ε4/ε4: OR 2.2, 95% CI = 0.7–6.7) (Farrer et al., 1997). Our study reports the *APOE* ε4 related risk for AD in a Taiwan Chinese population (ε3/ε4: OR 2.24, 95% CI = 1.76–2.84, ε4/ε4: OR 7.21, 95% CI = 3.11–16.76). When including our findings, the risk of having AD in *APOE* ε4 carriers was highest in Japanese, followed by Caucasian, Taiwan Chinese, African Americans and Hispanics. The comparison of *APOE* ε4 allele frequency in AD patients and the related risk for AD among different ethnicities is summarized in Table 6. This should be verified by more studies in the future.

**TABLE 6:** The comparison of *APOE* ε4 allele frequency in patients with Alzheimer’s disease and the related risk for Alzheimer’s disease among different ethnicities.

	*APOE* allele frequency,% in patients with AD			Odds ratio (95% confidence interval)*	
Ethnic group	ε 2	ε 3	ε 4	ε 3/ε 4	ε 4/ε 4
Caucasian	3.9	59.4	36.7	2.7 (2.2–3.2)	12.5 (8.8–17.7)
African American	7.7	59.1	32.2	1.1 (0.7–1.8)	5.7 (2.3–14.1)
Hispanic	6.3	74.5	19.2	2.2 (1.3–3.4)	2.2 (0.7–6.7)
Japanese	2.7	69.5	27.8	5.6 (3.9–8.0)	33.1 (13.6–80.5)
Taiwan Chinese	4.6	77.5	17.9	2.2 (1.8–2.8)	7.2 (3.1–16.8)

*APOE ε3/ε3 as reference. The data on the Caucasian, African American, Hispanic, and Japanese ethnicities are referenced from Farrer et al. (1997). AD, Alzheimer’s disease; *APOE*, apolipoprotein E; ε, epsilon.

Unlike previous studies (Payami et al., 1994; Farrer et al., 1997; Altmann et al., 2014), *APOE* ε4 carrier women and men did not show a significant difference in AD risk. This finding is consistent with a previous study that men and women with one copy of *APOE* ε4 have nearly the same odds of developing AD across the age span of 55–85 years (Neu et al., 2017). *APOE*ε4 homozygous women appear to have a increased risk compared to homozygous men, which aligns with the study conducted by Farrer et al. (1997). Their study showed that the OR for APOE ε4/ε4 leaps to 10 and above for men and women, but even among the homozygotes there appears to be a slightly greater effect in women (Farrer et al., 1997). However, it is important to interpret the result cautiously due to the limited number of *APOE* ε4 homozygotes in our study participants.

Our observation of a stronger effect of *APOE* ε4 in individuals under the age of 75 is consistent with previous reports that *APOE* ε4 exerts its maximal effect on AD risk by the early 70’s, with a reduction in risk after the age of 85 (Jarvik et al., 1995; Farrer et al., 1997). This finding is in line with epidemiological evidence that *APOE* ε4 is not only associated with an increased risk of the development of AD but also a lower age of onset (Corder et al., 1993; Rebeck et al., 1993; Farrer et al., 1997; Liu et al., 2013). The mean age of clinical onset is 68 years in ε4 homozygotes, 76 years in ε4 heterozygotes, and 84 years in ε4 non-carriers, indicating that *APOE* ε4 dramatically increases the risk of AD development with an earlier age of onset in a gene dose-dependent manner (Corder et al., 1993; Rebeck et al., 1993).

In our study, *APOE* ε4 carrier men showed a non-significant higher risk after the age of 75, whereas *APOE* ε4 carrier women exhibited an increase in risk before the age of 75. The increased risk of AD in women before the age of 75 years may be associated with the events that occur 15–20 years earlier, possibly coinciding with the period of menopause (Dubal and Rogine, 2017), which on average begins at 51 years of age, and during which physiological changes and estrogen loss occurs (McKinlay et al., 1992). A possible explanation for the higher AD risk in *APOE* ε4 carrier men at an older age is that the age-adjusted period of prevalence for cardiovascular disease (CVD) related to *APOE* genotype is higher in men than in women (18.6% in the ε4 group for men and 9.9% for ε4 women) (Lahoz et al., 2001). The prevalence of CVD has also been shown to increase with age (Yazdanyar and Newman, 2009; North and Sinclair, 2012), and individuals with CVD are at a higher risk for AD especially if they carry the *APOE*4 allele (Stampfer, 2006; Eriksson et al., 2010; de Bruijn and Ikram, 2014). Therefore, older men with *APOE* ε4 may have a higher risk of AD. This hypothesis needs further validation. It is also important to carefully exclude cognitive impairments caused by stroke or cerebrovascular diseases, as the comorbidity of CVD and AD is high in the elderly.

There were some limitations to the current study. First, we did not adjust for known AD risk factors, such as the number of years of education, family history of AD and vascular risk factors (Barnes and Yaffe, 2011), as they are all known risk factors for developing AD, in addition to *APOE* ε4. Second, no participants with *APOE* ε2/ε4 genotyping were recruited in our study. Therefore, whether the protective effect of the ε2 allele overcomes the risk brought on by the ε4 allele is unknown. Third, these findings are based on a population from hospital outpatient clinics and may not be generalized to the general population. Fourth, we did not report the prevalence of CVD in the recruited subjects; future studies with information on the prevalence of CVD are warranted to better understand the association between *APOE* genotype, CVD, dementia and AD, especially in older men. Even so, our findings warrant further investigation as it is likely a complex set of risk factors associated with AD development, and consideration should be given to age and sex-specific treatments for cognitive decline and AD. For example, if women are at an increased risk of AD at a younger age, it is plausible that treatments for women, especially those who carry an *APOE* ε4 allele, may need to be initiated earlier. Additionally, it is important to vigorously control modifiable cardiovascular risk factors, particularly in men who are *APOE* ε4 carriers, due to their higher risks of AD and CVD at an older age.

In conclusion, we reported the association between *APOE* genotype and AD in a Taiwan Chinese population. At older age (≥75 years), *APOE* ε4 carrier men have a higher risk of AD, while *APOE* ε4 carrier women have an increased risk at a younger age. This is important to consider for individual patients in terms of diagnostics, treatment, and genetic counseling.

## Data availability statement

The original contributions presented in this study are included in the article/supplementary material, further inquiries can be directed to the corresponding author.

## Ethics statement

The studies involving humans were approved by the Kaohsiung Medical University Hospital Institutional Review Board [KMUHIRB-SV(II)-20190059, KMUHIRB-SV(II)-20200034, KMUHIRB-E(II)-20220263, and KMUHIRB-SV(I)-20230025]. The studies were conducted in accordance with the local legislation and institutional requirements. Written informed consent for participation in this study was provided by the participants’ legal guardians/next of kin.

## Author contributions

L-CH and Y-HY contributed to the study conception and design. M-YL, C-FC, Y-PC, K-YL, and Y-HY contributed to the acquisition of data. L-CH, C-FC, Y-PC, and Y-HY analyzed and interpreted the data. L-CH and Y-HY conducted the statistical analyses and involved in writing the initial draft of the manuscript. All authors reviewed and revised the manuscript and approved the submitted version.
